# Long term follow-up of refractory/relapsed hairy cell leukaemia patients treated with low-dose vemurafenib between 2013 and 2022 at the Department of Internal Medicine and Oncology, Semmelweis University

**DOI:** 10.3389/pore.2023.1611378

**Published:** 2023-11-08

**Authors:** Kata Ferenczi, Zsófia Flóra Nagy, Ildikó Istenes, Hanna Eid, Csaba Bödör, Botond Timár, Judit Demeter

**Affiliations:** ^1^ Department of Internal Medicine and Oncology, Semmelweis University, Budapest, Hungary; ^2^ 1st Department of Pathology and Experimental Cancer Research, Semmelweis University, Budapest, Hungary

**Keywords:** relapsed/refractory, infection, hairy cell leukemia, BRAF inhibitors, vemurafenib

## Abstract

**Introduction:** Hairy cell leukemia (HCL) is an indolent B-cell lymphoproliferative disease. *BRAF* V600E mutation is detected in nearly all classical HCL cases which offers the possibility of targeted therapy.

**Objective:** The aim of our study was to assess the efficacy of low-dose vemurafenib as well as to assess the long term outcome of HCL patients treated with this drug at the Department of Internal Medicine and Oncology at Semmelweis University.

**Methods:** We report on 10 patients with classical HCL treated with low-dose vemurafenib at our Department between 2013 and 2022.

**Results:** As a result of fixed time low-dose vemurafenib treatment, 5 of 10 patients (5/10) achieved partial remission, 4 (4/10) had stable disease, and 1 (1/10) had MRD positivity. No patients achieved complete remission. The median progression-free survival was 28.5 months while the overall survival was 82 months.

**Conclusion:** We confirm that low dose of vemurafenib is effective and safe in the vast majority of patients with HCL. This small-molecule oral treatment allows to gain valuable time—months or even years—before further, usually parenteral treatment options have to be given or before previous treatment has to be repeated. There are also promising data supporting the combination of vemurafenib with other drugs for the treatment of HCL patients which could provide even further possibility to bridge treatment.

## Introduction

Hairy cell leukemia (HCL) is an indolent B-cell lymphoproliferative disease characterized by pancytopenia, splenomegaly, and infiltration of bone marrow, liver, and spleen with mature B cells. HCL as a well-defined entity was recognized in 1958 by [1].

It is a rare neoplasm representing 2% of lymphoid leukemias [2]. Affected patients often have non-specific symptoms including weakness and fatigue, as well as symptoms related to cytopenias and splenomegaly. Infectious complications are common, due to both the underlying immunosuppression from cytopenias and myelosuppressive therapy. In peripheral smear, the hairy cells present as characteristic-appearing mononuclear cells which are typically large with circumferential hair-like cytoplasmic projections and a round nucleus [3].

Diagnosis of HCL is based on the presence of hairy cells in the blood or bone marrow (BM). Hairy cells express the following markers: CD11c, CD19, CD20, CD22, CD103, CD123, and CD25. Histological examination of a bone marrow biopsy specimen is used to assess bone marrow infiltration. This highlights the clinical importance of immunophenotypic analysis. A wide range of antibodies have been reported as useful markers including annexin-A1, DBA.44 (mouse monoclonal HSL antibody), TRAP (tartrate-resistant acid phosphatase), Tbet, HBME1 (Hector Battifora) and BRAF V600E mutation specific mouse monoclonal antibodies [4].

The V600E mutation of the *BRAF* gene was identified in 2011 by using whole exome sequencing of genomic DNA from purified leukemic cells of a HCL patient by [5]. The mutation replaces thymine with adenine in exon 15 of *BRAF* at the 1799th nucleotide position of the coding sequence. This results in an amino acid change from valine to glutamate at the 600th amino acid position of the protein sequence and leads to aberrant activation of the MEK-ERK pathway, resulting in increased cell proliferation and survival. *BRAF* V600E mutation is detected in nearly all classical HCL cases and offers the possibility of targeted therapy [5]. The diagnostic toolkit has been expanded since 2011 (and is increasingly important) to detect the presence of the *BRAF* V600E mutation. *BRAF* gene mutation is detected by sequence analysis or using the monoclonal mouse antibody VE1 [6].

The current gold standard first-line treatment of hairy cell leukemia is the administration of purine nucleoside analogs (cladribine or pentostatin) [7]. Thanks to purine nucleoside analogue (PNA) treatment, the life expectancy of patients today does not differ from the life expectancy of the average healthy population, but 40% still have a relapse [8, 9]. Treatment with anti-CD20 monoclonal antibody, rituximab, is used with high efficacy not only in refractory cases but also in low tumor burden patients unsuitable for purine nucleoside analogue therapy [10–12]. Since the vast majority of HCL patients carry the *BRAF* p.V600E mutation the use of vemurafenib, a *BRAF* inhibitor, that shows an unusual specificity for the p.V600E mutation has been suggested in individual patients as early as 2012. By now it is an accepted treatment modality in relapsed/refractory hairy cell leukaemia patients. Moreover, in clinical studies the first line use of rituximab combined with vemurafenib is under evaluation.

## Methods and patients

We report the treatment characteristics and long-term outcomes of 10 classical HCL patients treated with vemurafenib between January of 2013 and December of 2022 at the 1st Department of Internal Medicine and Oncology, Semmelweis University. In our institution HCL patients were diagnosed and treated within the addressed 10 years. Early results of our vemurafenib treated patients have been published in our 2013 (these patients are marked as No. 1,2,3 in the current paper—same as in the 2013 publication) [3]. Over the past four decades in our center the majority of HCL patients have received first-line treatment with alfa interferon. If there were no contraindications to PNA treatment and after the improvement of the blood counts subcutaneous cladribine treatment followed. Since 2013 those elderly and frail patients who progressed despite treatment with alfa interferon have received second-line vemurafenib (Patients No. 7, 8 and 10 in the present paper). All the patients underwent dermatologic examination at baseline.

Clinical data and follow-up information were collected by regular follow-ups and chart review. Follow-ups and control visits were carried out during an active treatment biweekly, after the active treatment period monthly, and after 6 months of stable disease every 3 months. Responses were evaluated based on blood counts, bone marrow findings, and peripheral blood hairy cell count using standard criteria (Table 1).

**TABLE 1:** Criteria for response to treatment.

Response	Criteria
CR	ANC> 1.5 G/L, Hgb> 11 g/L, Thr> 100 G/L, no organomegaly by physical examination, no hairy cells in peripheral blood and bone marrow
MRD negative CR	ANC> 1.5 G/L, Hgb> 11 g/L, Thr> 100 G/L, no organomegaly, no hairy cell detected in bone marrow by immunohistochemistry
PR	ANC> 1.5 G/L, Hgb> 11 g/L, Thr> 100 G/L, organomegaly decreases by> 50%, hairy cell in peripheral blood <5%, bone marrow infiltration decreases by <50%
SD	CR and PR criteria are not met.
PD	Decrease in cell numbers> 25%, increase in organomegaly by> 25%
Relapse	Morphological relapse: recurrence of hairy cells in peripheral blood and/or bone marrow
	Haematological relapse: development of cytopenia involving at least one cell line

Patient characteristics are summarized in Table 2. Median age at diagnosis was 50 years (range, 35–68), at initiation of vemurafenib treatment was 73 years (range, 41–87). Patients were heavily pretreated (median of 2 prior treatment lines; range, 1–4 lines; *n* = 10). All consecutive treatments were administered due to progression.

**TABLE 2:** Data from our patients treated with vemurafenib.

Patient	Age at dg	Prior treatment lines	Time until treatment(month)	Duration of vemurfenib treatment (day)	Dose of vemurafenib (mg/day)	Response	PFS(month)	OS(month)	Side effect	Treatment after vemurafenib
1.	40	2 (caldribine, interferon)	12	58	2 × 240	PR	37	115	—	Rituximab, Cladribine
2.	45	3 (interferon, cladribine, rituximab)	180	14	2 × 240	SD	2	89	arthralgia hyperbilirubinaemia	Pentostatin
3.	65	3 (interferon, cladribine, rituximab)	96	110	2 × 240	SD	32	72	toxicoderma, arthralgia	IFN
4.	72	1 (interferon)	56	56	2 × 240	PR	32	32	photosensitivity	IFN
5.	64	2 (interferon, rituximab)	228	14	2 × 240	PR	49	49	tumorlysis sy (grade 2)	-
6.	48	3 (interferon, cladribine, rituximab)	72	91	2 × 240	SD	14	89	photosensitivity, arthralgia	IFN
7.	80	1 (interferon)	84	14	2 × 240	SD	25	24	toxicoderma	IFN
8.	69	1 (interferon)	96	56	2 × 240	PR	8	78	atrophy of skin	Rituximab
9.	35	4 (interferon, cladribine, interferon, rituximab)	216	47	2 × 480 for 28 days	PR	5	86	keratoacanthoma	Rituximab mono 4x
					2 × 720 until day 47					
10.	72	1 (interferon)	2	180	2 × 480	MRD	97	97	hyperkeratosis	—

Descriptive and comparative statistical methods were used to analyze the clinical data.

### BRAF V600E mutation–specific immunohistochemistry

Immunohistochemistry was performed on formalin-fixed, paraffin-embedded (FFPE) tissue using a mutation-specific antibody to BRAF V600E protein (VE1 clone; ready-to-use dilution; Ventana Medical System, Tucson, AZ) in a Leica Bond-Max automated immunostainer (Leica Biosystems, Deer Park, IL). Three-μm thick sections mounted on adhesive glass slides were deparaffinized and subjected to heat-induced epitope retrieval (HIER) at pH 9 (using Bond ER Solution 2) for 30 min before incubation with pre-diluted BRAF V600E mutation-specific primary antibody for 40 min. The Bond Polymer Refine Detection Kit (DS9800 Leica Biosystems, Deer Park, IL) was used to visualize reactivity. Immunoreactions were completed by nuclear counterstaining with hematoxylin.

### Molecular analysis for BRAF mutations

The mononuclear cell fraction of peripheral blood or bone marrow samples collected in EDTA containing collection tubes was separated by Ficoll gradient centrifugation. Cellular DNA was extracted using the MagCore Plus II Automated Nucleic Acid Extractor (RBC Bioscience Corporation, Taiwan). Isolated DNA samples were quantified using a Qubit 4 Fluorometer (Thermo Fisher Scientific, USA) and stored at 4°C. The extracted DNA was subjected to PCR amplification of a 91 bp region of the BRAF gene harboring codon V600 with a forward (TGA​AGA​CCT​CAC​AGT​AAA​AAT​AGG) and a biotin-conjugated reverse primer (TCC​AGA​CAA​CTG​TTC​AAA​CTG​AT) using the ABI-Veriti 96 Well Thermal Cycler (Thermo Fisher Scientific, Waltham, MA). The amplified sequence was subjected to pyrosequencing of codons 599 to 601 using PyroMark Q24 (Quiagen). Briefly, single-stranded DNA templates for were obtained with the assistance of the PyroMark Q24 Vacuum Prep Workstation (Qiagen) according to manufacturer’s instructions. The DNA template was incubated with the sequencing primer (5′-TGA​TTT​TGG​TCT​AGC​TAC​A-3′) on the PyroMark Q24 heat-block at 80°C–85°C for 2 min. Pyrosequencing was performed using the following dispensation order (CGATGATC) on the PyroMark Q24 system following the manufacturer’s guidelines. All sequencing results were confirmed by pathologist review.

## Results

Median time from diagnosis to treatment was 90 months (range, 2–228 months). Vemurafenib dose of 240 mg twice daily was used in 8 patients. Further patients received 480 mg twice daily (*n* = 2). Nine patients continued at this dose, whereas dose was escalated in 1 patient number 9. In patient number 9 we tried to deepen the remission by escalating the dose of vemurafenib. Dose and duration of the vemurafenib treatment was escalated in patient number 10 due to concomitant *M. tuberculosis* infection which was diagnosed before starting vemurafenib treatment. Median duration of vemurafenib treatment in the whole patient cohort was 56 days (range, 14–180 days).

Tables 3, 4 contains data on organ involvement, bone marrow infiltration and blood counts before initiating and after completion of vemurafenib treatment in all of our patients. As a result of vemurafenib treatment, 5 of 10 patients (5/10) achieved partial remission. 4 (4/10) had stable disease, and 1 (1/10) had MRD positivity. Patient number 1 achieved MRD positive CR after 180 days of vemurafenib treatment, at his 6 months bone marrow biopsy histology 2.4% residual hairy cells were found. MRD negative complete remission was not achieved in any of the patients. Median progression-free survival (PFS; from start of vemurafenib treatment to retreatment or death) was 28.5 months. Median overall survival (OS; from start of vemurafenib treatment to the end of our retrospective analysis or death) was 82 months. Median overall survival from the diagnosis to the end of the study or death was 164.5 months. During this long follow-up time 7 of 10 patients are still alive.

**TABLE 3:** Data about the blood counts, bone marrow- and organ involvement in our patients before vemurafenib treatment.

Patients	Before vemurafenib treatment				
-	-	-	Blood cell counts		
-	Bone marrow infiltration	Organ involvement	White blood cell (G/L)	Hemoglobin (g/L)	Platelet count (G/L)
1.	30% hairy cell	Hepatosplenomegaly	0.8	110	109
2.	80% hairy cell	Splenomegaly	0.8	100	49
3.	30%–40% hairy cell	—	1.1	110	107
4.	90% hairy cell	—	2.82	105	191
5.	diffuse infiltration	—	1.11	63	23
6.	80% hairy cell	Splenomegaly	2	147	71
7.	60%–70% hairy cell	—	11	75	44
8.	80% hairy cell	Splenomegaly	3.58	131	54
9.	40% hairy cell	—	2.47	129	90
10.	40% hairy cell	—	2.66	156	94

**TABLE 4:** Data about the blood counts, bone marrow- and organ involvement in our patients after vemurafenib treatment.

Patients	After vemurafenib treatment						
-	-	-	Blood cell counts				
-	Bone marrow infiltration	Organ involvement	White blood cell (G/L)		Hemoglobin (g/L)		Platelet count (G/L)
1.	<10% hairy cell	Splenomegaly	4		155		98
2.	no data	Splenomegaly	1.21		111		77
3.	40% hairy cell	—	2.3		141		118
4.	70% hairy cell	—	4.38		126		172
5.	there is no data	—	2.56		78		89
6.	40% hairy cell	—	3		159		112
7.	no data	—	2.77		104		72
8.	60% hairy cell	Splenomegaly	4.37		150		65
9.	20% hairy cell	—	3		111		162
10.	24% hairy cell	—	2.61		151		132

**FIGURE 1:**
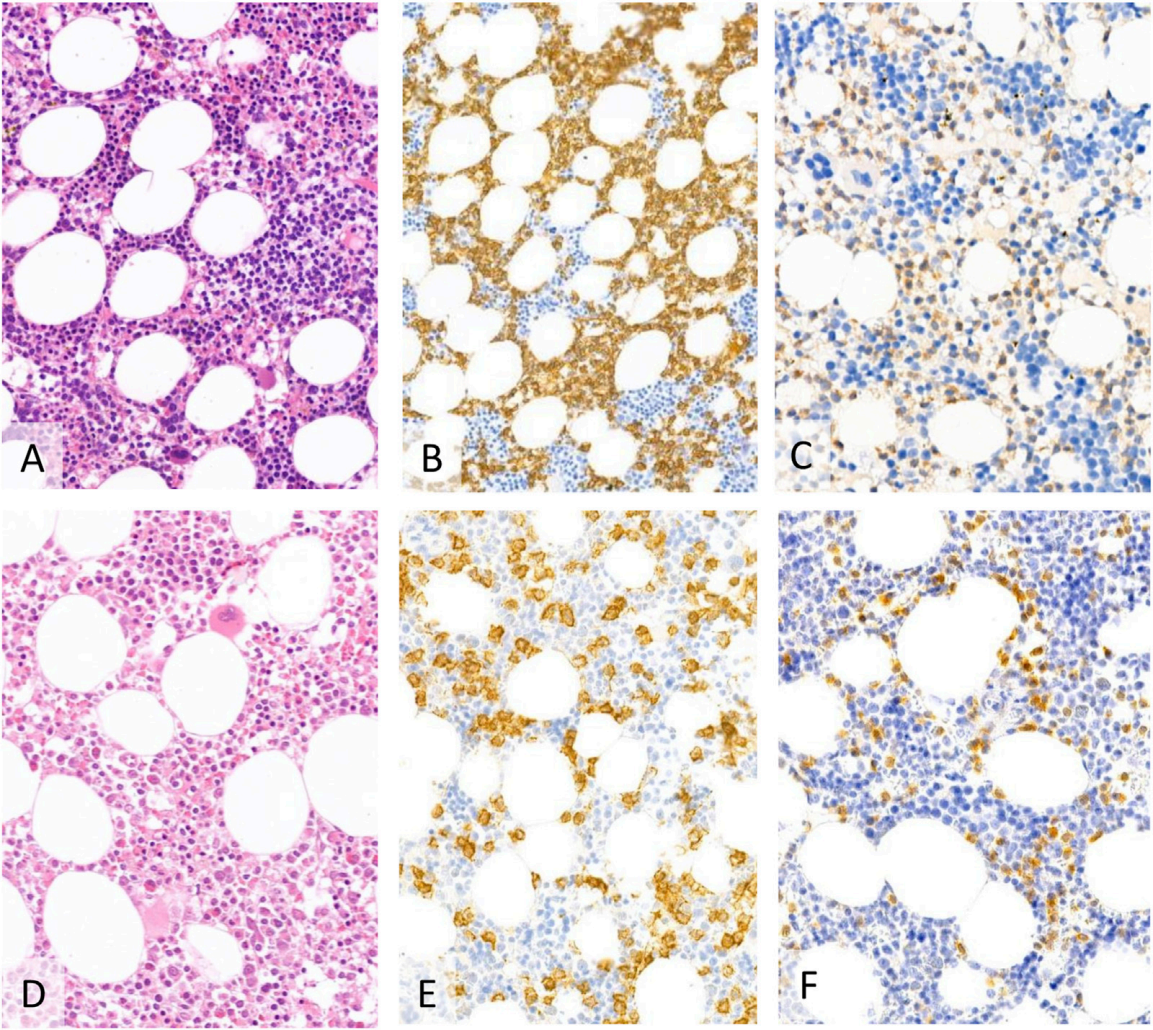
The first biopsy showed a more extensive infiltration (40%–50%) of hairy cell leukaemia (**(A)**–H&E, **(B)**–CD20, **(C)**–BRAF, 20X). After treatment the bone marrow was normocellular with maturing hematopoesis with residual patchy, interstitial lymphocytic aggregates (**(D)**–H&E) of CD20 positive **(E)** B-cells and with characteristic BRAF VE1 expression **(F)** accounting for ∼15%–20% of the cellularity.

Figure 1 shows the effectivity of vemurafenib exemplified by a patient’s control bone marrow biopsy.

To treat relapse following vemurafenib, 4 of 10 (4/10) patients received alpha interferon, 2 of 10 (2/10) patients received cladribine and rituximab, 1 of 10 patient (1/10) received rituximab, and 1 of 10 (1/10) received pentostatin. 2 patients did not receive treatment, because it was not necessary.

The side-effects of vemurafenib included photosensitivity (*n* = 2), toxicoderma (*n* = 2), keratoacanthoma (*n* = 1), hyperkeratosis (*n* = 1), arthralgia (*n* = 3), and elevation of bilirubin level (*n* = 1). In patient No. 5 grade 2 tumorlysis syndrome occurred as a side effect of the vemurafenib treatment within a few days. In patients with toxicoderma the skin symptoms disappeared after topical steroid treatment. Arthralgia was tolerable with non-steroidal agents. All patients were advised to stay out of the Sun. Side effects did not necessitate lowering of the dose or stopping the administration of vemurafenib.

## Discussion

The discovery of the activating mutation *BRAF* V600E in 2011 has provided novel insights into the pathogenesis and had implications for the diagnosis and targeted therapy of HCL. This disease-defining genetic mutation, detected in nearly 100% of HCL patients, has opened the opportunity to investigate new targeted agents for the treatment of HCL [5]. The first reports documented in HCL patients treated with vemurafenib, a BRAF inhibitor approved for first-line treatment of unresectable or metastatic melanoma, been published in 2012 [13]. BRAF inhibitors such as vemurafenib are currently not approved in HCL and are used “off-label” in patients with refractory/relapsed HCL [14] In two phase 2 studies in 2015, that enrolled 54 BRAF-mutated HCL patients relapsed/refractory to purine analogue-based treatments, vemurafenib was administered at the standard melanoma dose of 960 mg twice daily for 16–18 weeks, respectively [15]. In comparison, patients in our study only received vemurafenib for an average of only 8 weeks. The ORR was 100% in the US study, 96% in the Italian study and the CR rate was 42% and 35%, respectively. MRD was detectable in all CR patients and relapses occurred soon after treatment. In our study no patient achieved complete remission with the shorter and lower dosing schedule. However, the median PFS in the Italian study was 9 months, which is significantly lower than the PFS we reported in the current analysis [15].

Dietrich et al. published a retrospective analysis of 21 refractory/relapsed HCL patients treated with vemurafenib at a lower dose (240 mg/day) with a median follow-up time of 17 months. The median duration of treatment was 90 days. The CR rate was 40%. Interestingly the median PFS (17 months) of these 21 R/R HCL patients is very similar to that found in our retrospective analysis (28.5 months), although our patients did not achieve complete remission [16].

Tiacci et al published the highly efficient and well-tolerated combination of vemurafenib + rituximab in refractory/relapsed HCL. Thus, new potential chemotherapy-free agents, the combination of BRAF inhibitors and anti-CD20 monoclonal antibodies may improve the duration of response and may add to the therapeutic armamentarium [17]. However, compared to this combination treatment, the advantage of vemurafenib monotherapy is that it can be used orally, requires less monitoring and adverse events are low-grade and manageable. Combined inhibition of BRAF and MEK represents another promising chemotherapy-free regimen that avoids infusion-related reactions, and infections potentially associated with rituximab treatment. There are also promising preclinical data with B cell lymphoma 2 (Bcl 2) inhibitor venetoclax, which support combinations with other drugs for the treatment of HCL patients [18].

Our results show that low dose of vemurafenib is effective and safe in the vast majority of HCL patients. Although none of our HCL patients achieved CR, still the overall survival of our R/R HCL patients is excellent. This indicates the very good therapeutic potential of vemurafenib to bridge the time between more aggressive therapies. Thanks to the lack of myelosuppressive and immunosuppressive effects, unlike PNA, vemurafenib is a chemotherapy-free option to treat HCL patients who have an active infection and who cannot wait for the infection to clear before starting treatment for HCL [19]. Thus, it could be administered even during the COVID-19 pandemic or in the event of an impending pandemic, either in the first-line setting or as a bridge to PNA until the resolution of the infection [20].

## Data Availability

The original contributions presented in the study are included in the article/supplementary material, further inquiries can be directed to the corresponding author.
